# Digital Health Interventions in Older Adult Populations Living With Chronic Disease in High-Income Countries: Protocol for a Scoping Review

**DOI:** 10.2196/49130

**Published:** 2024-03-28

**Authors:** Mir Nabila Ashraf, Natasha L Gallant, Cara Bradley

**Affiliations:** 1 Centre on Aging and Health University of Regina Regina, SK Canada; 2 Department of Psychology and Centre on Aging and Health University of Regina Regina, SK Canada; 3 Dr John Archer Library and Archives University of Regina Regina, SK Canada

**Keywords:** chronic disease, high-income countries, digital health, interventions, older adults, quality of life

## Abstract

**Background:**

Globally, around 80% percent of adults aged 65 years or older are living with at least 1 chronic disease, and 68% percent have 2 or more chronic diseases. Older adults living with chronic diseases require greater health care services, but these health care services are not always easily accessible. Furthermore, the COVID-19 pandemic has resulted in unprecedented changes in the provision of health care services for older adults. During the COVID-19 pandemic, digital health interventions for chronic disease management were developed out of necessity, but the evidence regarding these and developed interventions is lacking.

**Objective:**

In this scoping review, we aim to identify available digital health interventions such as emails, text messages, voice messages, telephone calls, video calls, mobile apps, and web-based platforms for chronic disease management for older adults in high-income countries.

**Methods:**

We will follow the Arksey and O’Malley framework to conduct the scoping review. Our full search strategy was developed following a preliminary search on MEDLINE. We will include studies where older adults are at least 65 years of age, living with at least 1 chronic disease (eg, cancer, cardiovascular disease, chronic obstructive pulmonary disease, and diabetes), and residing in high-income countries. Digital health interventions will be broadly defined to include emails, text messages, voice messages, telephone calls, video calls, mobile apps, and web-based platforms.

**Results:**

This scoping review is currently ongoing. As of March 2023, our full search strategy has resulted in a total of 9901 records. We completed the screening of titles and abstracts and obtained 442 abstracts for full-text review. We are aiming to complete our full-text review in October 2024, data extraction in November 2024, and data synthesis in December 2024.

**Conclusions:**

This scoping review will generate evidence that will contribute to the further development of digital health interventions for future chronic disease management among older adults in high-income countries. More evidence-based research is needed to better understand the feasibility and limitations associated with the use of digital health interventions for this population. These evidence-based findings can then be disseminated to decision-makers and policy makers in other high-income countries.

**International Registered Report Identifier (IRRID):**

DERR1-10.2196/49130

## Introduction

Globally, according to the World Health Organization (WHO), 41 million people die from chronic diseases each year [1]. Four chronic diseases, including cancer, cardiovascular diseases, chronic respiratory diseases, and diabetes, are responsible for over 80% of all premature mortality worldwide [1]. Chronic disease outcomes are worse for older adults compared to their younger counterparts [2]. For instance, according to the US Centers for Disease Control and Prevention (CDC), around 85% of older adults in the United States endure at least 1 chronic disease and approximately 60% have at least 2 chronic diseases [3,4]. In Canada, approximately 70% of people aged at least 65 years are living with at least 1 of 10 common chronic diseases [4]. Life expectancy continues to increase, especially in high-income countries [5], and, given that age increases the risk of developing chronic diseases [6], the prevalence of chronic disease is also expected to grow. Thus, it is crucial to identify potential strategies for older adults with chronic diseases in high-income countries. Furthermore, while older adults living with chronic diseases require a greater amount of health care services, these health care services are not always easily accessible for this population [7].

The COVID-19 pandemic has had and continues to have a detrimental impact on the global health systems [8]. During the initial stages of the COVID-19 pandemic, older adults were often unable to access health care services due to public health mandates [9]. In the later stages of the COVID-19 pandemic, access to health care services was still limited due to lengthened waitlists and increased staff shortages [10]. Older adults living with chronic diseases and their caregivers experienced increased levels of stress and worse chronic disease outcomes due to this lack of access [11,12]. It is therefore essential that alternatives to in-person interventions be developed to ensure accessible health care services during public health emergencies such as the COVID-19 pandemic.

Amid the emergent COVID-19 pandemic, digital health interventions such as telehealth and app-based health care were developed in response to limitations to in-person interventions [13]. There are few evidence-based guidelines on digital health intervention for chronic disease management. Thus, this review is essential to identify available digital health interventions to manage chronic diseases among older adults. So, the primary aim of this scoping review is to identify available digital health interventions for chronic disease management among older adults in high-income countries. The secondary aim of this scoping review will be to identify potential outcomes associated with these digital health interventions such as early diagnosis, increasing health literacy for chronic disease management, or improving quality of life. Findings from this scoping review will further support the development and evaluation of future digital health interventions.

## Methods

### Overview

**Figure 1:**
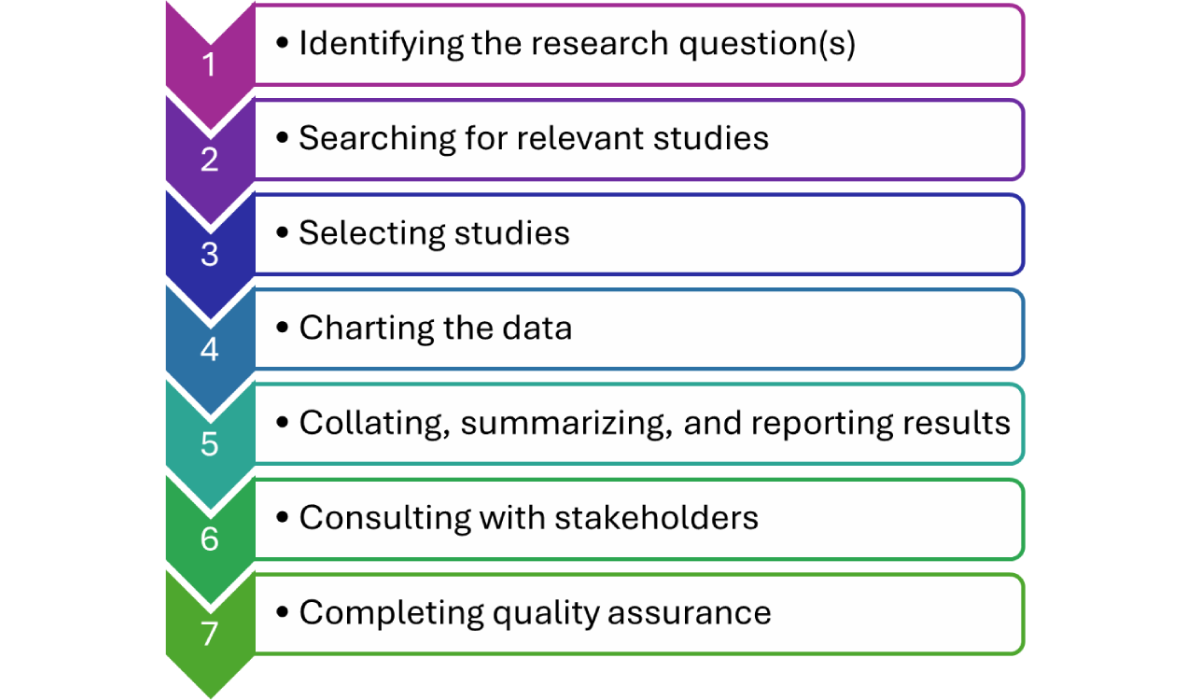
Arksey and O’Malley’s methodological framework with the additional step of quality assurance.

The 6 steps of the Arksey and O’Malley [14] framework will be used to conduct a scoping review to investigate digital health interventions to manage chronic disease among older adults in high-income countries. Given the possibility of a high risk of bias when conducting reviews [15], we will include a quality assessment as an additional step in our scoping review as recommended by Levac et al [16]. Thus, the scoping review will be completed in the 7 steps illustrated in Figure 1.

### Step 1: Identifying the Research Questions

For this scoping review, the research questions that have been identified are as follows: What are the digital health interventions aimed at improving outcomes associated with chronic disease management for older adults? (2) With regards to the identified digital health interventions, what are the associated outcomes (such as early diagnosis, prevention, or treatment for chronic disease or increasing health literacy on chronic disease management) for (1) older adults, (2) their caregivers, and (3) the health system?

### Step 2: Searching for Relevant Studies

A preliminary search was conducted in the Cochrane Database of Systematic Reviews, Joanna Briggs Institute (JBI) Evidence Synthesis, Open Science Framework, and International Prospective Register of Systematic Reviews (PROSPERO) to identify other similar scoping reviews. Following this preliminary search, the full search strategy was conducted by an academic librarian. An initial MEDLINE search was undertaken in February 2023 to identify papers on the topic by focusing on the concepts of older adults, caregivers, the health care system, digital interventions, and chronic disease. The text words contained in the titles and abstracts of relevant papers, as well as the index terms used to describe the papers were used to develop a full search strategy for Ovid MEDLINE(R) and In-Process, In-Data-Review and Other Non-Indexed Citations and Daily (1946-2023; Multimedia Appendix 1). Papers published in any language from database inception to the present were included. The search strategy, including all identified text words and index terms, was translated to CINAHL Plus (Ebsco; 1966-2023), JBI EBP Database (Ovid; 2012-2023), PsycINFO (Ovid; 1806-2023), and Web of Science (1966-2023) by the academic librarian that can be accessed upon request. Finally, using similar text words and index terms, sources of grey literature searched included Google Advanced, Grey Literature Report, and ProQuest Dissertations & Theses: Global (1861-2022). The full search strategy was undertaken on April 3, 2023.

### Step 3: Selecting Studies

Following a pilot test, titles and abstracts of studies including methodology of randomized controlled trials, nonrandomized controlled trials, pre-post studies, interrupted time-series studies, prospective cohort studies, retrospective cohort studies, case-control studies, and cross-sectional studies will be imported into Covidence (Veritas Health Innovation). The titles and abstract will be screened by 2 independent reviewers for assessment against the inclusion criteria for the scoping review as outlined below for full-text review. The selected papers will be downloaded for full-text review in detail against the inclusion criteria by 2 independent reviewers. Reasons for exclusion of full-text studies that do not meet the inclusion criteria will be recorded and reported in the scoping review. Any disagreements that arise between the reviewers at each stage of the selection process will be resolved through discussion or with a third reviewer.

### Inclusion Criteria

#### Population

The population of interest includes adults who are aged at least 65 years and living with chronic disease. For a study to be included in this review, at least 50% of the sample must be aged at least 65 years or the mean age of the sample needs to be at least 65 years. Chronic diseases considered eligible for this review include cancer, cardiovascular diseases, chronic obstructive pulmonary disease, and diabetes. Finally, studies will be included if they are with populations residing in 1 of the selected countries with a high or very high Human Development Index [17]—Australia, Canada, and the United States.

#### Interventions

Studies examining digital health interventions for chronic disease management will be included. Digital health interventions will be broadly defined to include emails, text messages, voice messages, telephone calls, video calls, mobile apps, and web-based platforms. Chronic disease management will be defined as the management of at least one of the following chronic diseases: cancer, cardiovascular diseases, chronic obstructive pulmonary disease, and diabetes.

#### Comparisons

Studies with comparisons to digital health interventions (such as placebo or health service delivery as usual) or without comparison (no comparative intervention), will be considered.

#### Outcomes

Following the initial screening of papers, we have identified a few potential outcomes such as studies that include outcomes related to physical health (eg, blood pressure and blood sugars) and mental health (eg, anxiety and depression) assessed with validated tools or approaches will be considered for this scoping review. Improvements in health education or health literacy as well as quality of life will also be considered as appropriate. However, we are open to adding other outcomes following full-text review after iterative discussion with research team members.

### Exclusion Criteria

The exclusion criteria for this study are (1) studies published in a form other than a peer-reviewed journal paper; (2) studies written in a language other than English; (3) studies with less than 50% of the sample aged at least 65 years or the mean age of the sample less than 65 years; (4) studies focused solely on chronic diseases other than cancer, cardiovascular diseases, chronic obstructive pulmonary disease, and diabetes; (5) studies published in a country other than Australia, Canada, or the United States; and (6) studies examining health interventions that are not digital (eg, in-person counseling) or digital interventions that are not focused on health (eg, general time management skills).

### Step 4: Charting the Data

Data will be extracted from studies included in the scoping review by 2 independent reviewers using a data extraction tool developed by the reviewers. Any disagreements that arise between the reviewers will be resolved through discussion or with a third reviewer. The data extracted will include the following details: authors, year of publication, objectives, type of study, population, intervention, comparisons, and outcomes. Where required, authors of papers will be contacted to request missing or additional data. A draft of our data extraction tool is provided (Multimedia Appendix 2). The draft data extraction tool will be modified and revised as necessary during the process of extracting data from each included paper. Modifications will be detailed in the full scoping review.

### Step 5: Collating, Summarizing, and Reporting the Results

The results will be reported in full in the final scoping review and presented in a PRISMA (Preferred Reporting Items for Systematic Reviews and Meta-Analyses) flow diagram [18,19]. When summarizing, we will list the type and format of interventions included in eligible studies. We will also organize similar outcomes together to determine the direction of outcomes included in the interventions. We will use descriptive numerical summary analysis for the results. The data will be mapped and presented in tabular format to make it easier to answer our research questions. A narrative summary will accompany the results and will describe how the results relate to the research questions.

### Step 6: Consultation With Stakeholders

For this step, we will consult with researchers from high-income countries with expertise in older adults, chronic disease management, and digital interventions to provide any relevant studies aligned with our research questions. We will also consult with a group of patient partners living with chronic disease to validate the study findings.

### Step 7: Quality Assessment

We will assess the quality of the studies included in this scoping review using JBI’s Critical Appraisal Checklist for Systematic Reviews and Research Syntheses [20]. Prior to critical appraisal, 2 independent reviewers will meet to determine the criteria for indicating a “yes,” “no,” “unclear,” or “not applicable” response in the context of this scoping review for each of the 11 items. For items that are deemed “unclear,” authors of the reviews will be contacted to request missing or additional data for clarification. Each study will be ranked according to findings from the critical appraisal as low quality (ie, “yes” for 0%-33% of critical appraisal items with a “yes” or “no”), medium quality (ie, “yes” for 34%-66% of critical appraisal items with a “yes” or “no”), and high quality (ie, “yes” for 67%-100% of critical appraisal items with a “yes” or “no”). The results of critical appraisal will be reported in a critical appraisal table.

## Results

Screening of titles and abstracts has been completed. The full-text review is expected to be complete by the end of October 2024, followed by data extraction in November 2024, and data synthesis in December 2024. We will disseminate the findings of the scoping review at the national and international scientific conference proceedings and by publishing the paper in a peer-reviewed journal.

## Discussion

### Principal Findings

With our rapidly aging population, digital health interventions may play a crucial role, especially for the management of chronic diseases among older adults. To our knowledge, our research will be one of the first that will highlight the available digital health interventions for the management of chronic disease which leads to major issues such as disability and interruption in the activities of daily living among older adults [7]. This scoping review will provide a comprehensive synthesis of existing digital interventions that will contribute to designing a feasible intervention model that can be customized by communities that need to manage chronic disease and improve the quality of life among older adults.

Though we will conduct a comprehensive scoping review, there may be some limitations that we will not be able to address. In this scoping review, we will only include peer-reviewed journal papers, so we may miss and exclude some of the evidence-based unpublished data from our findings. Another important limitation is that papers written in a language other than English will be excluded from our scoping review. Thus, additional research focusing on the available unpublished data findings and non-English papers would be helpful for generating evidence to gather available digital health interventions for chronic disease management.

The findings of the scoping review will assist researchers in the further establishment of digital health interventions for chronic disease management among older adults. More evidence-based research is crucial to understanding the potential barriers and enablers of digital health for older adults and, especially, older adults with chronic diseases.

### Conclusions

Digital health interventions have become a prevalent form of health care services since the COVID-19 pandemic, but more evidence-based research is needed to assess the outcomes associated with digital health interventions. Findings from this scoping review will provide an overview of empirical evidence regarding digital health interventions and provide a stepping stone for the development and evaluation of future digital health interventions for older adults living with chronic disease.

## Data Availability

Data sharing is not applicable to this article as no data sets were generated or analyzed during this study.

## Appendix

Multimedia Appendix 1 Database and search strategy.

Multimedia Appendix 2 Data extraction instrument.

## Abbreviations

CDC: US Centers for Disease Control and Prevention
JBI: Joanna Briggs Institute
PRISMA: Preferred Reporting Items for Systematic Reviews and Meta-Analyses
PROSPERO: International Prospective Register of Systematic Reviews
WHO: World Health Organization
