# Supplementary material for: Digital Health Interventions in Older Adult Populations Living With Chronic Disease in High-Income Countries: Protocol for a Scoping Review
Source: JMIR Res Protoc. 2024 Mar 28;13:e49130. doi: 10.2196/49130 (PMC11009846; doi:10.2196/49130)
Supplement: Multimedia Appendix 1 [file resprot_v13i1e49130_app1.docx]

Ovid MEDLINE(R) and In-Process, In-Data-Review & Other Non-Indexed Citations and Daily <1946 to February 14, 2023>

**Search Strategy:**

1 exp Aged/ 3436260

2 (Aging or aged or geriatric* or gerontol* or senior* or elder* or older or pensioner* or 65+).ab,kw,ti. 1868135

3 1 or 2 4583476

4 exp Chronic Disease/ 607122

5 exp Noncommunicable Diseases/ 2858

6 exp Cardiovascular Diseases/ 2683716

7 exp Neoplasms/ 3795112

8 exp Diabetes Mellitus/ 497905

9 (chronic diseas* or chronic condition* or hypertens* or diabet* or cancer* or neoplasm* or chronic pulmonary obstructive diseas* or COPD or cardiovascular diseas* or noncommunicable diseas* or non-communicable diseas*).ab,kw,ti. 3633146

10 4 or 5 or 6 or 7 or 8 or 9 8076087

11 exp Telemedicine/ 43291

12 exp Mobile Applications/ 11020

13 exp Internet/ 95870

14 (ehealth or e-health or electronic health or telehealth or mhealth or m-health or phon* or telephon*OR text message* or email* or voicemail or voice messag* or technolog* or virtual* or video call* remote or video call* or zoom or mobile app* or digital* or internet or web-based or smart phon* or smartphone*).ab,kw,ti. 1141658

15 11 or 12 or 13 or 14 1205730

16 exp Canada/ 179537

17 exp Australia/ 167668

18 exp United States/ 1452682

19 (Canad* or australi* or USA or united states or American*).ab,kw,ti. 1037963

20 16 or 17 or 18 or 19 2323563

21 3 and 10 and 15 and 20 7918

22 limit 21 to yr="2013 - 2023" 4927
