# Supplementary material for: Digital Health Interventions in Older Adult Populations Living With Chronic Disease in High-Income Countries: Protocol for a Scoping Review
Source: JMIR Res Protoc. 2024 Mar 28;13:e49130. doi: 10.2196/49130 (PMC11009846; doi:10.2196/49130)
Supplement: Multimedia Appendix 2 [file resprot_v13i1e49130_app2.doc]

Data extraction for all studies selected for critical appraisal will be completed using the following

categories:

1. Authors
2. Title
3. Journal
4. Year of Publication
5. Countries
6. Objective(s)
7. Population by Age, Sex, Ethnicity, Religion, and Other Characteristics (e.g., type of chronic diseases)
8. Study Methods including Randomized Controlled Trials, Non-Randomized Controlled Trials, pre-post Studies, Interrupted Time-Series Studies, Prospective Cohort Studies, Retrospective Cohort Studies, Case-Control Studies, and Cross-Sectional Studies
9. Intervention
10. Details of interventions (if applicable)
11. Outcomes (if applicable)
12. Details of these outcomes (e.g., how measured, if applicable)
13. Key findings that relate to the scoping review question(s)
14. Conclusions that relate to the scoping review questions
